# Perinatal Outcomes in Women with Chronic Kidney Diseases

**DOI:** 10.1055/s-0042-1753546

**Published:** 2022-12-29

**Authors:** Marcus Vinicius Pinheiro Zilli, Anderson Borovac-Pinheiro, Maria Laura Costa, Fernanda Garanhani Surita

**Affiliations:** 1 Department of Obstetrics and Gynecology, Universidade Estadual de Campinas, Campinas, SP, Brazil

**Keywords:** kidney disease, high-risk pregnancy, antenatal care, perinatal outcomes, doença renal, gravidez de alto risco, cuidado pré-natal, cuidados perinatais

## Abstract

**Objective** To assess maternal and neonatal outcomes in women with chronic kidney disease (CKD) at a referral center for high-risk pregnancy.

**Methods** A retrospective cohort of pregnant women with CKD was followed at the Women's Hospital of Universidade Estadual de Campinas, Brazil, between 2012 and 2020. Variables related to disease etiology, treatment duration, sociodemographic variables, lifestyle, other associated diseases, obstetric history, and perinatal outcomes were assessed. The causes of CKD were grouped into 10 subgroups. Subsequently, we divided the sample according to gestational age at childbirth, as preterm and term births, comparing maternal and neonatal outcomes, and baseline characteristics as well as outcomes among such groups.

**Results** A total of 84 pregnancies were included, in 67 women with CKD. Among them, six pregnancies evolved to fetal death, five to miscarriage, and one was a twin pregnancy. We further analyzed 72 single pregnancies with live births; the mean gestational age at birth was 35 weeks and 3 days, with a mean birth weight of 2,444 g. Around half of the sample (51.39%) presented previous hypertension, and 27.7% developed preeclampsia. Among the preterm births, we observed a higher frequency of hypertensive syndromes, longer maternal intensive care unit (ICU) stay in the postpartum period, higher incidence of admission to the neonatal ICU, higher neonatal death, lower 5-minute Apgar score, and lower birth weight.

**Conclusion** This study demonstrates increased adverse outcomes among pregnancies complicated by CKD and expands the knowledge on obstetric care among such women in an attempt to reduce maternal risks and identify factors related to prematurity in this population.

## Resumo

**Objetivo** Avaliar os desfechos maternos e neonatais em mulheres com doença renal crônica (DRC) em um centro de referência para gestação de alto risco.

**Métodos** Coorte retrospectiva de gestantes com DRC acompanhadas no Hospital da Mulher da Universidade Estadual de Campinas, Brasil, entre 2012 e 2020. Variáveis relacionadas à etiologia da doença, duração do tratamento, variáveis sociodemográficas, estilo de vida, outras doenças associadas, história obstétrica, número de consultas de pré-natal e os resultados perinatais foram avaliados. As causas da DRC foram agrupadas em 10 subgrupos. Posteriormente, dividimos a amostra de acordo com a idade gestacional no parto, pois os nascimentos pré-termo e a termo comparam os desfechos maternos e neonatais bem como as características basais e desfechos entre esses grupos.

**Resultados** Um total de 84 gestações foram incluídas em 67 mulheres com DRC. Dentre elas, seis gestações evoluíram para óbito fetal, cinco para aborto espontâneo, e uma era gestação gemelar. Foram analisadas ainda 72 gestações únicas, com nascidos vivos; a idade gestacional média ao nascer foi de 35 semanas e 3 dias, e o peso médio ao nascer foi 2.444 g. Cerca de metade da amostra (51,39%) apresentava hipertensão prévia e 27,7% desenvolveram pré-eclâmpsia. Entre os casos de prematuridade (34 casos), observamos maior frequência de síndromes hipertensivas, mais dias de internação materna na UTI no pós-parto, maior incidência de internação na UTI neonatal, óbito neonatal, menor índice de Apgar de 5 minutos e menor peso ao nascimento.

**Conclusão** Este estudo demonstra o aumento de desfechos adversos em gestações complicadas por DRC e amplia o conhecimento sobre cuidados obstétricos entre essas mulheres na tentativa de reduzir os riscos maternos e identificar fatores relacionados à prematuridade nessa população.

## Introduction

Chronic kidney disease (CKD) is a global health problem that affects ∼ 10% of the population. The prevalence has increased in recent decades and is higher among low- and middle-income countries. 1 In Brazil, more than ten million people have CKD. Chronic kidney disease occurs in women and men equally, and reproductive function can be affected in women, in addition to influencing maternal and neonatal outcomes. 2

Approximately 3 to 4% of women of reproductive age and ∼ 1 to 3% of pregnant women have CKD, regardless of the underlying cause. 3 4 In these patients, there is a greater risk of maternal hypertensive complications, fetal growth restriction, and premature birth; therefore, there is a greater chance of hospitalization of the newborn in a neonatal intensive care unit (ICU), stillbirth and neonatal death, in addition to morbidities related to prematurity. 5 Women with CKD are 10 times more likely to develop preeclampsia than women at usual risk, with a reported prevalence of preeclampsia of up to 40% among pregnant women with CKD. 6

The reported overall prevalence of preterm birth (before 37 weeks) in Brazil is around 10 to 12%. In other countries, rates vary according to other health indicators. One of the factors that can influence this is pregestational creatinine levels. A Canadian study of 56,000 pregnancies showed an increase in therapeutic preterm birth in women with pregestational creatinine above the 95 ^th^ percentile (0.87 mg/dL), which was not observed in patients with spontaneous preterm births. In this same study, a graph was constructed that illustrates a J-curve supporting the association of serum creatinine and the probability of preterm delivery, with a 1.23-fold increase in the chance of preterm delivery in patients who had some renal dysfunction compared with pregnant women with normal renal function. 7

Despite the possible unfavorable perinatal outcomes in pregnant women with CKD reported in the literature, there is still a lack of Brazilian studies on the subject. The aim of this study is to evaluate the maternal and perinatal outcomes of women with CKD who underwent prenatal care and delivery at a single Brazilian reference center for high-risk pregnancies, and further compare cases with preterm and term childbirth.

## Methods

We performed a retrospective cohort study at the Women's Hospital of Universidade Estadual de Campinas, Brazil, a referral university hospital in southeast Brazil, accounting for a surrounding population of 3,100,000 inhabitants. This study was approved by the research ethics committee of the institution (CAAE report 15429419.5.0000.5404).

We included all pregnancies of women with a previous diagnosis of CKD who underwent prenatal follow-up at the specialized antenatal care (ANC) outpatient clinic and who gave birth at the Women's Hospital between 2012 and 2020. All patients with high risk of preeclampsia were given prophylaxis with low dose aspirin and calcium supplementation, as recommended by institutional protocol. We collected data from the medical records on an electronic system by completing a data collection form specifically created for the study.

We evaluated variables related to CKD etiology, duration of kidney disease treatment, sociodemographic variables, lifestyle variables, other associated diseases, and obstetric history and perinatal outcomes. In the case of patients with more than one pregnancy during the study period, each index pregnancy was considered, that is, the unit of study was the pregnancy. The data obtained were entered into a database created for this study, in Excel format, which was reviewed to identify inconsistencies. The underlying causes of kidney disease were later grouped into 10 subgroups according to similar characteristics and frequency of diagnoses.

To describe the profile of the sample according to the variables under study, frequency tables of categorical variables were made with absolute (n) and percentage (%) frequency values, and descriptive statistics of numerical variables, with mean values and standard deviation. Subsequently, women who had a viable pregnancy (excluding abortions and fetal deaths) were divided into 2 groups according to the occurrence or not of prematurity (gestational age [GA] < 37 weeks). To compare the categorical variables between pregnancies that ended in preterm birth, the chi-squared test or Fisher exact test (for expected values lower than 5) were used.

The significance level adopted for the statistical tests was 5%. The software used was the SAS System for Windows version 9.2. (SAS Institute Inc, 2002–2008, Cary, NC, USA). 8 9 10 11

All Strengthening the Reporting of Observational Studies in Epidemiology (STROBE) requirements for an observational study were followed and verified in this article.

## Results

A total of 84 pregnancies were included, in 67 women with CKD who underwent absolute neutrophil count (ANC) between 2012 and 2020. Of these, 6 pregnancies evolved with fetal deaths and 5 with abortion, totaling 11 gestational losses, which corresponds to 13.1% of this sample. A diamniotic dichorionic twin pregnancy occurred in a 32-year-old primigravid patient with a history of systemic lupus erythematosus (SLE), CKD on dialysis, kidney transplant in 2010 and viral infection with loss of the transplanted kidney. The patient progressed to preterm labor at 32 weeks and underwent a cesarean section; the newborns were born weighing 1,460 g and 1,197 g, with favorable neonatal outcomes. Considering the single pregnancies that progressed to childbirth ( *n* = 72), the mean age of the pregnant women was 28.58 years (standard deviation [SD] = 6.34), with a mean time since diagnosis of CKD of 10.61 years (SD = 8.82). Most of the women were white, were in a stable relationship, + and had high school education; none of the participants reported using alcohol or illicit drugs. Table 1 shows the sociodemographic data of the patients included in the study.

**Table 1:** Characteristics of women with chronic kidney disease and singleton pregnancy that progressed to childbirth

	N = 72	%
Age (years)		
< 20	3	4.17
20–29	35	48.61
30–39	31	43.05
≥ 40	3	4.17
Age (mean)		
28.58 years		
Marital status		
Single	26	36.11
Stable relationship	46	63.89
Occupation*		
Paid work	11	15.28
Unpaid work	28	38.89
Schooling**		
Elementary	13	13.06
High school	33	45.83
University	5	6.94
Skin color***		
White	46	63.89
Non-White	24	33.33
Smoking	2	4.17
Number of Pregnancies		
1	31	43.05
2	16	22.22
≥ 3	25	34.73
Previous miscarriage		
0	59	81.94
≥ 1	13	18.06

Frequency missing *33 **21 ***2.

**Fig. 1:**
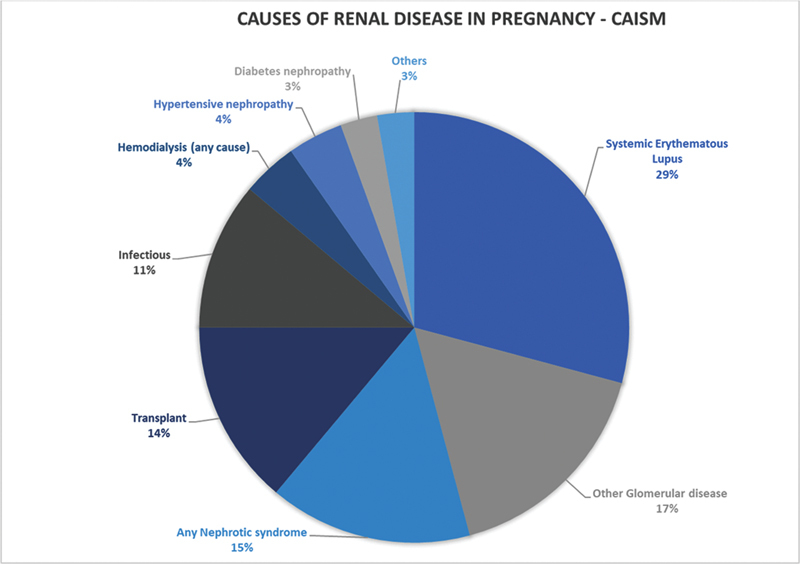
Main causes of chronic kidney disease among pregnancies followed at high-risk antenatal care ( *n* = 84).

We grouped the main causes of CKD into 10 categories, presented from the most frequent to the least frequent: SLE ( *n* = 21), glomerulopathy ( *n* = 12), nephrotic syndrome ( *n* = 11), transplant ( *n* = 10), infection ( *n* = 8), dialysis ( *n* = 3), hypertension ( *n* = 3), diabetes ( *n* = 2), and other diseases with lower frequency that were included in the “others” group ( *n* = 2: one patient with Wegener granulomatosis and the other with rheumatoid arthritis and nephrolithiasis). Fig. 1 shows the cause of CKD of the patients included in the study.

Among the 72 pregnancies that resulted in live births, the mean gestational age at birth was 35 weeks and 3 days (SD = 7.21), with a mean birth weight of 2,443.7 g (SD = 722.48). The majority had a birth at term (52.78%) or late preterm (34.72%). Eight newborns were classified as small for gestational age (11.1%), and 5 of them were children of mothers with SLE (⅝ = 62.5%). Five children were classified as low birth weight newborns (0.69%). Table 2 shows obstetric and neonatal data for patients with CKD included in the study. These women attended an average of 9.29 (SD = 3.88) prenatal consults. Approximately a quarter (20/72; 27.78%) of the study population developed preeclampsia during pregnancy.

**Table 2:** Obstetric history, data on pregnancy, childbirth, and neonatal outcomes of women with chronic kidney disease

	N = 72	%
Hypertensive syndrome		
Chronic hypertension	17	23.61
Chronic hypertension with superimposed preeclampsia	12	16.67
Preeclampsia	8	11.11
Without hypertension	35	48.61
Mode of birth		
Vaginal	23	31.94
C-Section	49	68.06
Maternal morbidities after birth*		
Hemorrhage	6	8.33
Infection	4	5.56
Others****	6	8.33
None	53	76.61
Maternal ICU admission**		
Yes	9	12.50
No	62	86.11
Apgar score at the first minute		
< 7	14	19.44
≥ 7	57	79.17
Apgar score at the fifth minute**		
< 7	3	4.17
≥ 7	68	94.44
Gestational age at birth		
GA < 28	4	5.55
≥ 28 GA < 32	5	6.95
≥ 32 GA < 37	25	34.72
GA ≥ 37	38	52.78
Neonatal ICU admission**		
Yes	26	36.11
No	45	62.50
Neonatal death***		
Yes	6	8.83
No	62	86.11

Abbreviation: GA, gestational age; ICU, intensive care unit.

Missing *3 **1 ***4 (no information due to medical transfer of the newborn to another hospital) ****Others: one hypoglycemia, four with hypertensive spikes, one with hypervolemia and dialysis.

Considering the high prevalence of preterm birth among the considered cases, and the burden of this condition for mothers and their children in the short and long term, we aimed to investigate conditions associated with this event. We compared the two groups according to the occurrence or not of preterm birth. There was a significant association between the occurrence of premature birth (gestational age < 37 weeks) and the need for a woman to be hospitalized in the ICU after childbirth, a higher occurrence of complications after childbirth, and a greater number of days of hospitalization ( Table 3 ). The majority cause of preterm birth among our population was preeclampsia (12/34 = 35.3%) and premature rupture of membranes (4/34 = 11.7%). This population had more prevalence of ICU admission and more days staying in the hospital.

**Table 3:** Comparison of maternal characteristics and outcomes according to the occurrence of preterm birth ( *n* = 72)

Variables	Preterm birth ( *n* = 34)	Term birth ( *n* = 38)	*p* -value
Maternal age (mean / SD)	29.03 (6.44)	28.18 (6.31)	0.560 *
Years since diagnosis (mean / SD)	10.00 (7.68)	11.13 (9.77)	0.894*
Maternal hospitalization in days (mean / SD)	4.85 (2.93)	3.13 (1.44)	0.001*
Group of kidney disease			0.143**
SLE	8 (23.5%)	13 (34.2%)	
Glomerular disease	4 (11.7%)	8 (21.1%)	
Nephrotic syndrome	5 (14.7%)	6 (15.8%)	
Transplant	4 (11.7%)	6 (15.8%)	
Infectious	7 (20.6%)	1 (2.6%)	
Hemodialysis	2 (5.9%)	1 (2.6%)	
Hypertensive nephropathy	2 (5.9%)	1 (2.6%)	
Diabetes nephropathy	2 (5.9%)	0	
Others	0	2 (5.3%)	
Skin color			0.892**
White	23 (67.3%)	23 (63.9%)	
Non-white	11(32.7%)	13 (36.1%)	
Hypertensive syndrome			0.009**
yes	23 (67.7%)	14 (36.8%)	
no	11 (32.3%)	24 (63.2%)	
Preeclampsia			0.178**
yes	12 (35.3%)	8 (21.0%)	
no	22 (64.7%)	30 (78.9%)	
Mode of birth			0.663**
vaginal	10 (29.4%)	13 (34.2%)	
cesarean	24 (70.6%)	25 (65.8%)	
Postpartum maternal ICU			0.006**
yes	11 (34.4%)	3 (11.9%)	
no	21 (65.6%)	35 (92.1%)	0.029**
Adverse maternal outcome #			
yes	11 (35.5%)	5 (13.2%)	
no	20 (64.5%)	33 (86.8%)	

Abbreviations: ICU, intensive care unit; SD, standard deviation; SLE.

* Kruskal-Wallis test, ** Qui-square test, *** Fisher test # adverse maternal outcome: bleeding, infection or other; #2 missing data.

## Discussion

The present study reports increased adverse maternal and neonatal outcomes among cases of CKD followed at a referral maternity hospital. Overall, around 13.1% of pregnancies progressed to abortion or stillbirth, and, among the cases of livebirths, almost half were preterm deliveries, with around one quarter complicated by preeclampsia. The cases of preterm delivery were associated with increased adverse outcomes, with 6 neonatal deaths.

According to international epidemiological data, the average rate of preterm births in the general population is 7 to 12% of births and, of these, ∼ 12% occur due to preeclampsia. 12 In our study, as expected, the incidence of premature births was much higher than in the general population (almost 50%), which is most likely due to CKD itself or secondary to the development of hypertensive syndromes and their consequences. The incidence of prematurity among pregnant women with CKD found in our study is similar to the data reported in the literature. A meta-analysis of 23 studies and 506,340 pregnant women concluded that CKD increased the risk of preeclampsia 10-fold, the risk of premature delivery and small-for-gestational-age newborns and led to a 3-fold increased risk of cesarean section. 6 Another study showed an association between CKD stage and its implications, with an increased incidence of prematurity as the CKD stage increased (CKD stage 1: 23.5% preterm; stage 2: 50.6%; stage 3: 78.4%; stage 4–5: 88.9%), using a serum creatinine threshold of 1.9 mg/dL, observed 93% newborn survival, 59% preterm birth, and fetal growth restriction of 37%. 13 14 In our study, we did not distinguish the CKD stage of the patients; however, our data are similar to the data from this study, since the incidence is within this range. Other studies have already shown that there is an association between CKD and prematurity, and one showed a 1.23-fold increase in relative risk of preterm birth in patients with prepregnancy kidney dysfunction, compared with those with normal renal function. 2 7 13 15

Our data show that prematurity was most likely associated with more severe cases, with a greater number of women hospitalized in adult ICUs among those who had premature births (around one-third of cases), while in patients with term birth, the number of ICU admissions was much lower (less than 10%). Maternal factors associated with CKD can increase the chance of patients being hospitalized in the ICU, due to the complexity of their cases, especially in those undergoing dialysis.

Epidemiologically, the most prevalent risk factors for CKD in the general population are arterial hypertension and diabetes mellitus; however, among pregnant women (mostly young women), other comorbidities are associated with the loss of renal function. 16 In our study, the mean age of the patients was 28.5 years, with multiple other causes for CKD. Additionally, the study hospital is the referral hospital for some diseases, including SLE.

It was also possible to verify that CKD was a risk factor for the development of hypertensive syndromes during pregnancy, more frequently observed in the group of patients whose outcome was preterm birth.

It is known that CKD is a factor for hypertensive syndromes, as observed in several studies. One study of 778 women with CKD reported that 25.3% presented chronic arterial hypertension, and the incidence of preeclampsia was 9.3%. 17 Another study reported an incidence of chronic arterial hypertension of 30.5% and of preeclampsia of 24.6%, with a higher rate of preeclampsia, explained in the study by almost a quarter of patients having CKD at more advanced stages (3–5). 18 In addition, meta-analyses and cohorts show that women with CKD are 10 times more likely to develop preeclampsia, and up to 40% of patients with preeclampsia have had CKD previously. 2 6 19 20 21 22

Acetylsalicylic acid has been recommended as an effective intervention to reduce the incidence of preeclampsia, especially in women with known risk factors, including those with CKD, preferably introduced between 12 and 16 weeks. 19 23 24 A study showed that its use may reduce the chance of developing preeclampsia and intrauterine growth restriction 24 , while another showed a reduction in the incidence of severe preeclampsia among patients with CKD stages 3 to 5, with no evidence in this study among patients with CKD stages 1 to 2. 18 24 However, another controlled trial showed a reduction in preterm preeclampsia in patients that used aspirin. 25 Our patients, guided by the institution protocol, used aspirin prophylaxis throughout the pregnancy, as well as calcium supplementation, as this is recommended in some groups of patients such as those with SLE. 26

The overall incidence of preeclampsia in the general population is ∼ 4.6 to 8.1%, depending on the region. 5 19 27 In this study group, we saw a rate of 27.78%, high compared with the general population, but close to the values found in other studies that evaluated populations with CKD. We currently know that CKD, even in its early stages, is associated with the production of proinflammatory cytokines, which triggers endothelial inflammation and consequently increases the chance of developing hypertension. In a normal pregnancy, there is a balance between angiogenic factors (among them PlGF and VEGF) and anti-angiogenic factors (sFlt-1), favoring good placental implantation. However, when there is an imbalance between these factors, poor implantation of the placenta or worsening of placental perfusion can occur, leading to a reduction in factors such as PlGF and VEGF and an increase in sFlt-1, causing endothelial inflammation and increasing the chances of developing preeclampsia. 5 Biomarker assessment may be an interesting way to adequately distinguish preeclampsia from other complications that can present with worsening proteinuria and hypertension. 28

In addition, there is evidence that these proinflammatory factors can cause glomerular damage, leading to proteinuria, which can worsen renal function or favor the development of preeclampsia. 15 29 Some of these studies report that the decline in kidney function is even worse among patients with CKD stages 3 to 5, while other studies did not see any worsening of renal function in stages 1 to 3. 15 18 These data in the literature still lacks consensus and require further investigation.

Our study presented some limitations. Given that our data was from a single center, it was not possible to further investigate the association between the reported adverse outcomes and the diverse CKD reported. In addition, pregnant women were referred to our service after the diagnosis of kidney disease, implying that we had no data related to the kidney biopsy, details about the infections that caused kidney failure or previous treatments. Our patients had a very heterogeneous treatment during pregnancy based on the causes and evaluation of their kidney disease. It was possible only to the group of women who underwent dialysis. The others received basic care to treat the underlying disease, such as hypertension, lupus, and diabetes. However, our sample is relevant to referral centers in a middle-income setting.

In our study, 68.06% of the patients underwent cesarean section, a number higher than that recommended by the World Health Organization (WHO) and higher than the Brazilian average. 30 31 However, in this case, these are patients with a greater possibility of complications, or acute or chronic fetal distress, which may be factors that increase the chances of opting for cesarean delivery. Even so, it is a high rate of cesarean sections, similar to that seen in other studies, which found cesarean section rates of 37 to 59%. 13 14 This corroborates the results of the study by Zhang et al., which showed a 3-fold increase in the number of cesarean sections in patients with CKD. 6

## Conclusion

These data reinforce that pregnancy complicated by CKD can present increased adverse maternal and perinatal outcomes, in addition to worsening the underlying disease or renal function. It is necessary to counsel these women on adequate family planning, to help plan their pregnancies when their kidney disease is stable and controlled. These are patients who require multiprofessional evaluation at a referral center, with special attention and care during high-risk prenatal care. With a planned pregnancy, it is possible to better evaluate risk factors and prognosis, and evaluate the indication of prophylaxis for preeclampsia, in addition to undertaking maternal-fetal surveillance and monitoring.
